# Supplementary material for: Case management interventions in chronic disease reduce anxiety and depressive symptoms: A systematic review and meta-analysis
Source: PLoS One. 2023 Apr 14;18(4):e0282590. doi: 10.1371/journal.pone.0282590 (PMC10104285; doi:10.1371/journal.pone.0282590)
Supplement: S2 File — (PDF) [file pone.0282590.s003.pdf]

SUPPLEMENTAL DATA I

PubMed search strategy

("Case Management"[Mesh] OR "Disease Management"[Mesh:NoExp] OR "Patient Care Management"[Mesh:NoExp] OR “Patient Care Planning”[Mesh:NoExp] OR "Patient-Centered Care"[Mesh] OR case manag*[tiab] OR care manag*[tiab] OR disease manag*[tiab] OR collaborated care[tiab] OR care coordination[tiab] OR coordinated care[tiab] OR integrated care[tiab] OR stepped care[tiab] OR managed care[tiab] OR patient centered care[tiab] OR patient centred care[tiab])

AND

("Anxiety Disorders"[Mesh] OR “Depressive Disorder”[Mesh] OR "Apathy"[Mesh] OR anxiety[tiab] OR anxious[tiab] OR depression*[tiab] OR depressed[tiab] OR depressive[tiab] OR apath*[tiab] OR "Accidental Falls"[Mesh] OR fall*[tiab] OR "Urinary Tract Infections"[Mesh] OR urinary tract infection*[tiab] OR "Deglutition Disorders"[Mesh:NoExp] OR deglutition[tiab] OR swallow*[tiab] OR "Hallucinations"[Mesh:NoExp] OR hallucinat*[tiab])

AND

("Pulmonary Disease, Chronic Obstructive"[Mesh] OR COPD[tiab] OR "Heart Failure"[Mesh] OR heart failure[tiab] OR "Diabetes Mellitus"[Mesh] OR diabetes[tiab] OR "Parkinson Disease"[Mesh] OR Parkinson*[tiab] OR "Alzheimer Disease"[Mesh] OR "Dementia"[Mesh] OR dement*[tiab] OR "Neoplasms"[Mesh] OR cancer[tiab] OR "Asthma"[Mesh] OR asthma[tiab] OR "Arthritis, Rheumatoid"[Mesh] OR rheumatoid arthritis[tiab] OR "Hypertension"[Mesh] OR hypertension[tiab] OR "Multiple Sclerosis"[Mesh] OR multiple sclerosis[tiab] OR chronic*[tiab]))

SUPPLEMENTAL DATA II

A. Cochrane Handbook for Systematic Reviews of Interventions: quality assessment of RCT

	**Random sequence generation** (selection bias)	**Allocation concealment** (selection bias)	**Blinding of participants and personnel** (performance bias)	**Blinding of outcome assessment** (detection bias)	**Incomplete outcome data** (attrition bias)	**Selective reporting** (reporting bias)	**Other sources of bias** (other bias)*
**Bogner et al. 2012**	+	+	+	+	+	+	+
**Callahan et al. 2006**	+	+	+	+	+	+	-
**Chen et al. 2018**	+	+	+	+	+	+	+
**Connor 2019**	+	+	+	+	+	+	+
**Egan et al. 2002**	+	+	?	-	+	?	+
**Ell et al. 2008**	+	?	+	+	+	+	+
**Gabbay et al. 2013**	?	?	?	+	-	+	+
**Gellis et al. 2012**	+	+	+	+	+	+	+
**Hernández et al. 2015**	+	+	?	?	+	+	+
**Kalter-Leibovici et al. 2017**	+	+	+	-	-	+	+
**Katon et al. 2010**	+	?	+	+	+	+	+
**Kroenke et al. 2010**	+	+	?	?	+	+	+
**Mertz et al. 2017**	+	+	?	-	+	+	+
**Miklavcic et al. 2020**	+	+	+	+	+	+	?
**Morgan et al. 2013**	+	-	-	-	+	+	+
**Riegel et al. 2006**	+	+	+	+	+	+	-
**Rose et al. 2017**	+	+	-	-	-	+	+
**Steel et al. 2016**	+	+	?	+	-	+	+
**Stoop et al. 2015**	+	+	?	?	?	+	+
**Titova et al. 2017**	-	-	?	?	+	+	-
**Tsuchihashi-Makaya et al.2013**	?	?	?	?	-	+	+
**Williams et al. 2004**	+	+	+	+	+	+	+
**Wu et al.**	?	?	?	?	+	+	+

Low risk of bias = + , high risk of bias = -, unclear risk of bias = ?

A. ROBINS-I tool quality assessment for non-randomized intervention studies

	Bias due to confounding	Bias in selection of participants into the study	Bias in classification of interventions	Bias due to deviations from intended interventions	Bias due to missing data	Bias in measurement of outcomes	Bias in selection of reported results	Overall risk of bias judgment
**Avci et al. 2019**	Serious	Low	Low	Moderate	Serious	Moderate	Low	Low
**Crowley et al. 2016**	Low	Moderate	Low	Low	Low	Moderate	Low	Low
**Johnson et al. 2014**	Serious	Low	Low	Low	Low	Moderate	Low	Low
**Lu et al. 2020**	Serious	Low	Moderate	Low	Low	Low	Low	Moderate

Low risk of bias, moderate risk of bias, serious risk of bias
