# Supplementary material for: Case management interventions in chronic disease reduce anxiety and depressive symptoms: A systematic review and meta-analysis
Source: PLoS One. 2023 Apr 14;18(4):e0282590. doi: 10.1371/journal.pone.0282590 (PMC10104285; doi:10.1371/journal.pone.0282590)
Supplement: Attachment — Submitted filename: KMM Manuscript systematic review Parkinsons Disease PLOS ONE.docx [file pone.0282590.s004.docx]

**Effectiveness of case management interventions in reducing common and potentially preventable complications associated with Parkinson’s disease:**

**A systematic review and meta-analysis**

Angelika D. van Halteren^1^, Jules M. Janssen Daalen^1^, Jan H.L. Ypinga^1^, Bastiaan R. Bloem^1^, Marjan J. Meinders^2^, Marten Munneke^1^, Sirwan K.L. Darweesh^1^

^1^ Radboud university medical center; Donders Institute for Brain, Cognition and Behaviour; Department of Neurology; Center of Expertise for Parkinson & Movement Disorders; Nijmegen, The Netherlands

^2^ Scientific Center for Quality of Healthcare, Radboud Institute for Health Sciences, Radboud university medical center, Nijmegen, The Netherlands

**Corresponding author**

Sirwan K.L. Darweesh, MD, PhD

Department of Neurology

Donders Institute for Brain, Cognition and Behaviour

Radboud university medical center

P.O. Box 9101 (internal code 914)

6500 HB Nijmegen

The Netherlands

Email: sirwan.darweesh@radboudumc.nl

**Word count abstract:** 349 words

**Word count article:** 3860 words (excluding tables and figures)

**Running title**: Effectiveness of case management interventions in reducing common and potentially preventable complications associated with Parkinson’s disease: A systematic review and meta-analysis

**Keywords**: care management, Parkinson’s Disease, integrated care

**Financial Disclosure/Conflict of Interest concerning the research related to the manuscript**

The Radboudumc Center of Expertise for Parkinson & Movement Disorder was supported by a center of excellence grant from the Parkinson’s Foundation.

Prof. Bastiaan Bloem currently serves as Editor in Chief for the *Journal of Parkinson’s disease*, serves on the editorial board of *Practical Neurology* and *Digital Biomarkers*, has received honoraria from serving on the scientific advisory board for Abbvie, Biogen, and UCB, has received fees for speaking at conferences from AbbVie, Zambon, Roche, GE Healthcare and Bial, and has received research support from the Netherlands Organization for Scientific Research, the Michael J Fox Foundation, UCB, Abbvie, the Stichting Parkinson Fonds, the Hersenstichting Nederland, the Parkinson’s Foundation, Verily Life Sciences, Horizon 2020, the Topsector Life Sciences and Health, the Gatsby Foundation and the Parkinson Vereniging.

Dr. Sirwan Darweesh was supported by a Parkinson’s Foundation- Postdoctoral Fellowship (PF-FBS-2026).

Angelika van Halteren, Jules Janssen Daalen, Jan Ypinga, Marjan Meinders and Marten Munneke have no financial disclosures or potential conflicts of interest to report.

**Funding sources of study**

This research is part of the PRIME project, funded by the Gatsby Foundation [GAT3676] and the Ministry of Economic Affairs, using the PPP Allowance made available by the Top Sector Life Sciences & Health to stimulate public-private partnerships. The Center of Expertise for Parkinson & Movement Disorders was supported by a center of excellence grant by the Parkinson Foundation.

**ABSTRACT**

**Background:** There is no systematic insight into the effect of case management on common complications associated with Parkinson’s disease (PD). This is a significant knowledge gap, given that people living with PD have identified care coordination as one of their highest priorities. Furthermore, it remains unclear whether the putative beneficial effects of case management would vary by crucial patient characteristics, such as their age, gender, or disease characteristics. Such insights would shift from “one size fits all” healthcare resource allocation to personalized medicine.

**Objective**: We sought to systematically examine the effectiveness of case management interventions on common preventable complications associated with PD, both in persons with Parkinson’s disease and in persons with other chronic health conditions. We specifically focused on falls, depression, anxiety, hallucinations, urinary tract infections, and swallowing impairments.

**Methods:** We identified studies published until November 2021 from PubMed and Embase databases using predefined inclusion criteria. For each study, data were extracted independently by two researchers. Narrative analysis, and when possible also, meta-analysis and random-effects analyses were undertaken.

**Results:** 23 randomized controlled trials and four non-randomized studies reported data on the effect of case management on feelings of anxiety (8 studies) or symptoms of depression (26 studies). Only one study was conducted on people with PD. No studies investigated the effects of case management on falls, hallucinations, urinary tract infections, or swallowing impairments. Across meta-analyses, we observed a statistically significant effect of case management on reducing anxiety (Standardized Mean Difference [SMD]= - 0.47; 95% confidence interval [CI]: -0.69, -0.32) and depression (SMD= - 0.48; CI: -0.71, -0.25). We found large heterogeneity in effect estimates across studies, but this was not explained by patient population or intervention characteristics.

**Conclusions**: Among people with chronic health conditions, case management has beneficial effects on symptoms of depression and feelings of anxiety, two common complications of PD. Currently, case management interventions in PD are rare. Future studies should assess the utility of case management for potentially preventative and common complications in people with Parkinson’s disease, focusing on the optimal content, frequency, and intensity of case management.

**1. INTRODUCTION**

The increasing prevalence of chronic disease poses a substantial burden to the capacity of health care services to provide adequate care [1]. In the case of Parkinson’s Disease (PD), the treatment requires continuous monitoring and ongoing interdisciplinary collaboration between health care providers of various disciplines, who ideally deliver proactive care [2]. However, current health care systems are typically designed to treat chronic diseases using a “one size fits all” approach instead of tailoring care to each patient’s individual needs [3, 4]. Consequently, persons with chronic disease diseases often become responsible for coordinating their care. As a result, patients receive fragmented and ineffective care in achieving the desired health outcomes [2, 4]. This increases health care costs and causes an unnecessary burden on patients and their carers, which, in turn, negatively affects their quality of life.

To address these challenges, case management has been introduced to improve the care coordination [5, 6]. Field defines case management in different ways[5, 7]. However, the everyday basis for each definition is that case management is a collaborative process involving one case manager or a small team that plans, coordinates and reviews the delivery of health care services to meet a patient’s individual needs [8]. According to this integrated care approach, a case manager takes over the responsibility of managing non-acute services. The interpretation of case management can vary substantially. Still, common core elements include the development and review of individualized care plans, organization of multidisciplinary case meetings, screening and monitoring of risk factors and symptoms, use of evidence-based guidelines, information support for involved physicians, empowerment of patients through providing education, and enhancing self-management skills [8-10].

There is emerging evidence for the promising effect of case management interventions on reducing hospital (re-)admissions and length of stay in patients with chronic diseases such as asthma [11, 12], diabetes [13, 14], chronic heart failure [14, 15] or established obstructive pulmonary diseases [16, 17]. However, the effect of case management on common complications associated with PD remains unknown. This is a significant knowledge gap, given that people living with PD have identified care coordination as one of their highest priorities. Furthermore, it remains unclear whether the putative beneficial effects of case management on common complications would vary by crucial patient characteristics, such as demographics (i.e., age or gender), disease severity or duration, or critical elements of the intervention (e.g., the number of individual patient contact). Such insights would facilitate a more comprehensive deployment of case management to susceptible subgroups of patients, thereby contributing to a broader shift from “one size fits all”-based healthcare resource allocation to “personalized” medicine.

To close these gaps in knowledge, we conducted a systematic review and meta-analysis to examine: 1) the extent and quality of evidence for the effectiveness of case management interventions on common potentially preventable complications associated with PD by using insights from other chronic diseases; and 2) to what extent putative effects of case management vary across patient subgroups.

**2. METHODS**

This systematic review is guided according to the PRISMA checklist (Preferred Reporting Items for Systematic Reviews and Meta-Analysis) [18].

***2.1 Search focus***

Knowing that an initial search that focused exclusively on case management interventions in people with PD yielded very few results, we expanded the scope of this review by including data on case management interventions in people with other chronic health conditions, in whom we hypothesized case management interventions would have similar effects as in people with PD. Specifically, we broadened the search by including all chronic diseases in which case management has been investigated: Alzheimer’s disease, asthma, cancer, chronic obstructive pulmonary disease (COPD), chronic heart failure, dementia, diabetes, hypertension, multiple sclerosis, and rheumatoid arthritis.

We focused our search on the following common potentially preventable complications associated with PD [19, 20] : (1) mood disorders, including depressive symptoms and anxiety; (2) fractures or injuries caused by falls; (3) swallowing impairment; (4) urinary tract infections; and (5) neuro-psychiatric disorders, including hallucinations.

***2.2 Search strategy***

An initial and limited search for empirical literature was undertaken by one reviewer [ADvH] in PubMed to identify important Medical Subject Headings (MeSH) and keywords describing relevant articles. Next, a systematic search for research-based literature was performed by two independent reviewers [ADvH, JMJD] using the identified MeSH terms and keywords. We exclusively focused on published articles referenced in PubMed and Embase online in July 2019. The following MeSH terms were used to identify studies on case management: “Case Management,” “Disease Management,” “Patient Care Management,” “Patient Care Planning,” and “Patient-Centered Care,” which were combined with relevant keywords. The detailed search strategy for PubMed can be found in Supplemental Data I. Finally, the reference lists of included studies were screened to identify studies missed by the search. A verification search was performed in November 2021.

***2.3 Selection criteria***

Abstracts and titles of all obtained studies were independently and systematically examined for the selection criteria by two reviewers [ADvH, JMJD], and disagreements were resolved during consensus meetings with a third reviewer [SKLD]. Search limits were applied to include only English articles and those published in a peer-review journal. We included in this systematic review studies that (1) used an observational (prospective and retrospective) or interventional study design; (2) included results on the association of a case management intervention with at least one of the five common and potentially preventable complications; (3) included populations who were diagnosed with one of the selected chronic diseases [aged ≥ 18 years]; and (4) described the case management intervention clearly and contained at least three core elements; (5) defined a clear control group, usually receiving usual care; and (6) reported at the minor measures of the distribution of age and gender for the intervention and control group, as potential confounders (Table 1). We excluded studies involving participants living in residential nursing homes, as those participants received 24-hour care and were thus not comparable to other populations receiving case management. Also excluded were studies that described only study protocols or conference abstracts.

***2.4 Data extraction and analysis***

The quality of the articles was evaluated by two authors (JMJD and ADvH) using the Cochrane risk of bias tool for randomized controlled trials (RCTs) [21] and the ROBINS-I tool for nonrandomized studies [22]. Two review authors [ADvH, JMJD] independently screened and evaluated the studies. The following data were extracted from each study: author, publication year, trial design, country, the aim of the study, study design, distribution of participant characteristics (sex, age, and diagnosis of disease), aspects of case management intervention (table 1), parts of care received by the control group, follow-up duration, study outcomes, and primary outcome results (mean, confidence intervals, standard deviation, standard error, interquartile range). Standard deviations (SD) were calculated from standard errors (SE). If no SD or SE was reported, authors were contacted and asked to provide additional data. We did not receive further data Field [23-27] for five articles, so these articles could not be included in the meta-analysis and random-effects regression analysis. We calculated standardized effect sizes for the main results of each study. Heterogeneity was assessed using the I^2^ statistics. We used funnel plot visualization and Egger’s test for funnel plot asymmetry to evaluate whether there was evidence for small-study effects.

We conducted meta-analyses using restricted maximum likelihood (REML) and DerSimonian-Laird estimator in a random-effects model because of significant heterogeneity between estimates across studies. In addition, predefined random-effects regression analyses were performed to identify effect modifiers of the effects of case management interventions if sufficient studies were available within a complication category (e.g., depressive symptoms, anxiety). Two studies were excluded from this analysis on depression, as the necessary data for regression analysis was not available [28, 29]. We included the following study population characteristics as potential effect modifiers: mean age, percentage of female participants, and disease group (neurodegenerative vs. internal medicine diseases). We also included the number of case management components (out of nine members, which are outlined in Table 1) as a potential effect modifier. Due to restrictions in the number of potential effect modifiers, we could not include individual components in the random-effects regression analyses for feelings of anxiety. Instead, we had the number of included case management components as potential effect modifiers. Multicollinearity of covariates – the modifying effect of covariates on each other - was assessed using variance inflating factors (VIF). We considered associations with p<0.05 to be statistically significant across meta-analyses and meta-regression analyses. Analyses were conducted in R [30], using packages meta for [31] and ggplot2 for visualization [32].

**3. RESULTS**

***3.1 Study selection***

The combined PubMed and Embase searches yielded 4765 unique records. After abstract review, 57 full-text articles were assessed for eligibility, of which 23 fulfilled our selection criteria. The other 34 were excluded for the following reasons: 25 studies did not provide relevant data on the topic under study; four studies were not identified as case management interventions; two studies dealt with a different health population; two studies did not include original data, and one study presented the same cohort. Field [28, 33] identified two further studies through cross-reference checking, and two studies, [26, 34], were added through verification search, bringing the total to 27 included studies. Figure 1 provides an overview of the search and study selection process.

***3.2 Study characteristics***

Table 2 describes the characteristics of the included studies. Of the 27 studies included, 23 [23-25, 27-29, 33-49] were RCTs and four were non-randomized intervention studies [26, 50-52]. The studies were published between 2002 and 2020 and evaluated case management interventions in various countries. The majority of studies evaluated case management interventions among patients with a single health condition [23-29, 35-38, 40-42, 44, 45, 47-52], whereas five studies [33, 34, 39, 43, 46] included patients with two or more different chronic diseases, such as heart failure and asthma or COPD. Only one of the included studies was conducted among people with PD. Taken together, the studies included 3752 participants ascribed to case management interventions. Mean age ranged from 57 years to 80 years, with an average age across all studies of 65 years, with 51% being female among the 25 studies [23-28, 33-52] reporting gender. Three thousand six hundred eighty-two participants were ascribed to the usual care group. The mean age ranged from 51 years to 78 years, with an average age across all studies of 51 years, with 51% being women.

***3.3 Risk of bias and quality assessment***

Of the four non-randomized intervention studies, three were rated as carrying a high risk of bias and one as moderate. Of the RCTs, only eight studies [23, 28, 33, 34, 36, 37, 39, 48] were judged to be of excellent methodological quality and at low risk of carrying bias. The remaining 15 studies were of low or moderate quality with a high or unknown risk of bias among several domains. However, most RCTs were rated as having severe or unfamiliar trouble on the parts of blinding participants and blinding of outcome assessment, which is less applicable to this kind of intervention. Details on the quality assessment are presented in Supplemental Data II.

***3.4 Components of case management intervention***

Across the 27 included studies, there was substantial heterogeneity across the content and duration of the various case management interventions. Table 3 displays the different strategies used in each study. Typical components of case management among the 27 studies were (1) regular telephone contacts combined with in-person visits; (2) monitoring of signs, symptoms, and risk factors; (3) ensuring therapy adherence; and (4) providing educational support on disease management and treatment or training on self-management skills. However, the content and structure of these components varied highly among these studies. For instance, in-person home visits ranged from developing and discussing a therapeutic plan with the patient in one study [40] to monitoring changes in signs and symptoms and reviewing patients’ safety in their home environment in another study [36]. The location of in-person visits also varied from the patient’s home to a clinical setting. Most studies were conducted through a combination of in-person and telephone contacts, with only seven studies [24, 26, 37, 41, 44, 45, 51] reporting an intervention exclusively conducted through telephone contact.

More commonly reported case management interventions, assisted with social and financial support, organized multidisciplinary case meetings and medication reviews, and developed individualized care plans. In only four studies, case management interventions also included the support of informal carers [24, 25, 34, 35]. And of these, only one study had a regular assessment of the carer’s physical health and education on the carer’s coping skills and educating carers in the disease management [35].

Furthermore, several studies incorporated technological support systems, which supported the implementation of case management strategies and offered new possibilities. For instance, a web-based service facilitates communication between the patient and the care team, schedules patient contacts, and keeps track of progress and current disease treatment field[35]. In a different study, a web-based collaborative intervention facilitated peer-to-peer support for patients with cancer through a chat room connecting all enrolled participants [29].

Of the 27 studies, 23 [24-27, 29, 33-44, 47-52] reported that case management interventions were delivered by a nurse case manager or a team consisting of a nurse case manager and other health care specialists. In one study, [28], the case manager was a depression clinical specialist without further clarification of the specialist's background. In another one, [23], two research coordinators fulfilled the role of care managers. In the other two studies, the background of the case manager was not specified any further by [45, 46]. The length of follow-up ranged from one and a half months to 24 months, with 18 studies reporting a 12-month or even longer follow-up period (Table 2).

***3.5 Overview of outcome measurements***

Results of the narrative data synthesis are summarized and presented in Table 2. Nearly all included studies evaluated the effectiveness of case management interventions on depressive symptoms, whereas anxiety was addressed in only eight studies [24, 27, 40, 42, 45-47, 50]. None of the included studies reported falls, urinary tract infections, swallowing impairment, or hallucinations.

***3.5.1 Effect of case management on anxiety***

Eight studies (n = 1239 participants) reported outcomes on symptoms of anxiety [24, 27, 40, 42, 45-47, 50]. The most commonly used scale was the Hospital Anxiety and Depression Scale (HADS) [24, 27, 40, 42, 45, 47], followed by 7-item Generalized Anxiety Disorder [46], and State Trait Anxiety Index (STAI) [50]. Six studies reported sufficient data and were included in a random-effects meta-analysis, the results of which revealed a significant effect of case management interventions in decreasing anxiety (Standardized Mean Difference [SMD]= - 0.47; 95% confidence interval [CI]: -0.69, -0.324 with moderate heterogeneity (I^2^ = 51.9%) (Figure 2).

Variance inflating factor (VIF) analysis showed no evidence of multicollinearity (all VIFs <5). A tendency for lower SMD was found for studies with a higher percentage of females [standardized regression coefficient β=0.01, p value=0.05]. No significant effect was found for mean age of intervention group [β= 0.02, p=0.13] and number of components were [β=0.13, p=0.21].

**3*.5.2 Effect of case management on depressive symptoms***

With the exception of one study [50], all studies (n=7314 participants) reported depressive symptoms measurements using a total of six depression scales, including the 9-item Patient Health Questionnaire (PHQ-9) [23, 25, 28, 36, 37, 39, 43, 44, 46, 49, 51, 52], the Hospital Anxiety/Depression Scale (HADS) [24, 27, 40, 42, 45, 47], the Center for Epidemiologic Studies Depression scale (CES-D) [29, 34, 38, 39], 20-item Symptom Checklist (SCL-20) [27, 33, 41, 49], the Cornell Scale for depression [35] and Taiwanese Depression Questionnaire [26].

Twenty studies reported sufficient data and were included in the meta-analysis (Figure 3). Two studies [39, 49] reported two different depression outcomes (CES-D/SCL-20 PHQ-9), but for analytical reasons, only one (PHQ-9) was included in the meta-analysis. A random-effects meta-analyses revealed a significant effect of case management intervention on depression (SMD= - 0.48; CI: -0.71, -0.25), but heterogeneity was high (I^2^ = 92.3%). A funnel plot and Egger’s test of funnel plot asymmetry (p = 0.57) showed no evidence for publication bias.

Variance inflating factor (VIF) analysis showed no evidence of multicollinearity (all VIFs<5). There were no significant population or disease characteristics: neurodegenerative diseases (reference: internal medicine diseases) [β=0.20, p = 0.80], mean age of intervention group [β= -0.01, p = 0.76] and percentage of females assigned to case management intervention [β=-0.01, p = 0.43]. Moreover, no significant effect was found for any of the individual case management components: development and review of individualized care plans [β= -0.60, p = 0.21]; in-person contact with the case manager [β=-0.09, p=0.89]; medication review [β= 0.39, p = 0.47]; provision of education on disease management and treatment [β=-0.08, p = 0.91]; self-management support [β=-0.24, p = 0.70]; support and training for healthcare providers [β=0.05, p = 0.92]; therapy adherence [β=0.73, p = 0.92] and use of evidence-based guidelines [β=0.27, p = 0.52].

**4. DISCUSSION**

This systematic review and meta-analysis show that case management is more effective than usual care at reducing depressive symptoms and anxiety, two common preventable complications associated with PD. This effect persisted for less complex case management interventions (whereby complexity was based on the number of included elements), across countries, for different chronic diseases and given the nearly equal representation of female and male participants across both genders. Contrary to our hypotheses, we found no evidence for effect modification by case management intervention, study population characteristics, or duration of follow-up. Our data showed that the beneficial effects of case management interventions on anxiety were somewhat more distinct in studies with a higher percentage of men and studies with a relatively old population. However, both observations were not statistically significant. No similar trends were observed for the effects of case management interventions on depressive symptoms. We did not identify any studies reporting on falls, hallucinations, swallowing impairment, and urinary tract infections, so the effect of case management on those complications remains unclear. Although we specifically looked at common complications in PD, only one of the included interventions was conducted in people with PD [37], indicating that case management interventions in PD are still rare. The randomized study reported that a PD nurse-led care management intervention among veterans led to better adherence to quality-of-care indicators. The screening instrument showed significant improvement among the intervention group compared to usual care [37]. In this trial, the PD nurse used four strategies to specify the PD problems of each veteran and to develop an action plan: (1) a telephone-administered assessment to identify 28 problem areas; (2) evidence-based care protocols and, if not available, use of expert consensus on care management; (3) patient portal for communication purposes; and (4) documentation templates to provide care that is patient-centered and coordinated. However, this study is limited by geographical and time factors.

Our study extends the findings of previous studies indicating a beneficial effect of case management on patients’ clinical health outcomes and functioning in everyday life [6, 53, 54]. While previous research on case management has focused on its effects on reducing hospital (re-)admissions, length of stay, and costs, little research has been done regarding its potential to reduce disease complications. Our systematic review addresses this gap in knowledge in a disease in which case management will likely have high potential. The findings of this systematic review favor case management interventions over usual care and suggest that even less complex case management interventions have a beneficial effect on common preventable complications of PD. Case management reduces feelings of anxiety and depressive symptoms, both complications that can have an immense impact on the quality of life in people with PD [55, 56]. Several indirect working mechanisms may have contributed to the beneficial effect on depressive symptoms and anxiety (figure 2), which can have an immense impact on the quality of life and even mortality in patients with PD [55-57]. First, patients across studies received personalized management of their chronic health conditions, including individually tailored health information and problem-solving strategies provided by a case manager. Second, the availability of one main contact person for newly arising issues and the ensuing establishment of a personal relationship between patients and case managers may have helped reduce anxiety and depressive symptoms. Notably, previous research on improving PD care revealed that having a single point of access was rated as the top priority by people with PD [3]. Third, having little information about the rate of disease progression and treatment options is known to enhance feelings of anxiety in patients. Patient education eventually allows for more shared decision-making and, thereby, a treatment that is better tailored to a patient’s individual needs and coping behavior, which, in turn, might alleviate anxiety.

Several methodological considerations need to be considered. First, heterogeneity across studies was high, and effect estimates varied substantially between studies, which might have affected the results of our meta-regression, leading to the inability to identify any covariates of our effect estimates. Furthermore, since only two studies [38, 47] followed patients for longer than two years, the long-term effectiveness of case management interventions remains largely unclear. Second, only a few included studies were of good methodological quality. In particular, limited blinding of outcome assessment, insufficient details on the specification of the efficacy of specific case management elements, and the lack of participant selection limited our ability to assess case management impact more accurately. In addition, as a systematic search strategy cannot screen full-text articles, the risk remains that the used search strategy does not capture relevant articles. For instance, Eggers et al. Field [58] conducted an RCT with a nurse case-manager-led intervention with patients with PD and reported on the effectiveness of CM in reducing hospitalization caused by falls. As these findings have not been reported in the study's abstract, this study was not captured through our search strategy.

Our data showed that the beneficial effects of case management interventions on anxiety were somewhat more distinct in studies with a higher percentage of men and studies with a relatively old population. However, both observations were not statistically significant. No similar trends were observed for the effects of case management interventions on depressive symptoms. This systematic review identified the need for further research into the most effective case management interventions and components, the optimal intensity and frequency of the individual case management strategies, and their interaction with patient characteristics. Once this knowledge becomes available, case management interventions can be implemented that are better tailored to individual needs and, as such, presumably more effective. Moreover, this systematic review revealed that case management implementation is more common among certain chronic diseases than others; the commonest ones were diabetes and heart failure, while only one included study concerned patients with PD. Future research is now warranted to (1) improve the current evidence base for case management effects on anxiety and depression in persons living with PD and (2) to provide additional insights regarding its effectiveness on other common PD complications.

**Author roles**

1. Research project: A. Conception, B. Organization, C. Execution; 2. Statistical Analysis: A. Design, B. Execution, C. Review and Critique; 3. Manuscript Preparation: A. Writing of the first draft, B. Review and Critique

Angelika D. van Halteren: 1B, 1C, 2A, 2B, 3A
Jules M. Janssen Daalen: 1B, 1C, 2A, 2B, 3A
Jan H.L. Ypinga: 3B
Bastiaan R. Bloem: 3B
Marjan J. Meinders: 3B
Marten Munneke: 3B
Sirwan K.L. Darweesh: 1A, 2C, 3B

**REFERENCES**

1.	Nugent R. Chronic diseases in developing countries: health and economic burdens. Ann N Y Acad Sci. 2008;1136:70-9. Epub 2008/06/27. doi: 10.1196/annals.1425.027. PubMed PMID: 18579877.

2.	van der Eijk M, Faber MJ, Al Shamma S, Munneke M, Bloem BR. Moving towards patient-centered healthcare for patients with Parkinson's disease. Parkinsonism Relat Disord. 2011;17(5):360-4. Epub 2011/03/15. doi: 10.1016/j.parkreldis.2011.02.012. PubMed PMID: 21396874.

3.	Vlaanderen FP, Rompen L, Munneke M, Stoffer M, Bloem BR, Faber MJ. The Voice of the Parkinson Customer. J Parkinsons Dis. 2019;9(1):197-201. Epub 2018/10/31. doi: 10.3233/JPD-181431. PubMed PMID: 30373962.

4.	Bloem BR, Henderson EJ, Dorsey ER, Okun MS, Okubadejo N, Chan P, et al. Integrated and patient-centred management of Parkinson's disease: a network model for reshaping chronic neurological care. Lancet Neurol. 2020 (in press).

5.	Lukersmith S, Millington M, Salvador-Carulla L. What is Case Management? A Scoping and Mapping Review. Int J Integr Care. 2016;16. doi: 10.5334/ijic.2477. PubMed PMID: WOS:000389673800002.

6.	Joo JY, Huber DL. An integrative review of nurse-led community-based case management effectiveness. Int Nurs Rev. 2014;61(1):14-24. Epub 2013/11/14. doi: 10.1111/inr.12068. PubMed PMID: 24218992.

7.	Lee DT, Mackenzie AE, Dudley-Brown S, Chin TM. Case management: a review of the definitions and practices. J Adv Nurs. 1998;27(5):933-9. Epub 1998/06/24. doi: 10.1046/j.1365-2648.1998.t01-1-00566.x. PubMed PMID: 9637319.

8.	Hudon C, Chouinard MC, Lambert M, Diadiou F, Bouliane D, Beaudin J. Key factors of case management interventions for frequent users of healthcare services: a thematic analysis review. Bmj Open. 2017;7(10). doi: ARTN e01776210.1136/bmjopen-2017-017762. PubMed PMID: WOS:000422617500195.

9.	Reilly S, Hughes J, Challis D. Case management for long-term conditions: implementation and processes. Ageing Soc. 2010;30:125-55. doi: 10.1017/S0144686x09990183. PubMed PMID: WOS:000274160800007.

10.	Hallberg IR, Kristensson J. Preventive home care of frail older people: a review of recent case management studies. J Clin Nurs. 2004;13(6B):112-20. Epub 2005/02/24. doi: 10.1111/j.1365-2702.2004.01054.x. PubMed PMID: 15724826.

11.	Chongmelaxme B, Lee S, Dhippayom T, Saokaew S, Chaiyakunapruk N, Dilokthornsakul P. The Effects of Telemedicine on Asthma Control and Patients' Quality of Life in Adults: A Systematic Review and Meta-analysis. J Allergy Clin Immunol Pract. 2019;7(1):199-216 e11. Epub 2018/07/29. doi: 10.1016/j.jaip.2018.07.015. PubMed PMID: 30055283.

12.	Burke H, Davis J, Evans S, Flower L, Tan A, Kurukulaaratchy RJ. A multidisciplinary team case management approach reduces the burden of frequent asthma admissions. ERJ Open Res. 2016;2(3). Epub 2016/10/13. doi: 10.1183/23120541.00039-2016. PubMed PMID: 27730207; PubMed Central PMCID: PMCPMC5034600.

13.	De La Rosa M, Pitts S, Chen PH. An interprofessional collaboration of care to improve clinical outcomes for patients with diabetes. J Interprof Care. 2019:1-3. Epub 2019/08/09. doi: 10.1080/13561820.2019.1643297. PubMed PMID: 31390908.

14.	McCants KM, Reid KB, Williams I, Miller DE, Rubin R, Dutton S. The Impact of Case Management on Reducing Readmission for Patients Diagnosed With Heart Failure and Diabetes. Prof Case Manag. 2019;24(4):177-93. Epub 2019/05/31. doi: 10.1097/NCM.0000000000000359. PubMed PMID: 31145236.

15.	Huntley AL, Johnson R, King A, Morris RW, Purdy S. Does case management for patients with heart failure based in the community reduce unplanned hospital admissions? A systematic review and meta-analysis. BMJ Open. 2016;6(5):e010933. Epub 2016/05/12. doi: 10.1136/bmjopen-2015-010933. PubMed PMID: 27165648; PubMed Central PMCID: PMCPMC4874181.

16.	van Eeden AE, van de Poll I, van Vulpen G, Roldaan T, Wagenaar W, Boland MRS, et al. Effectiveness of case management in the prevention of COPD re-admissions: a pilot study. BMC Res Notes. 2017;10(1):621. Epub 2017/11/28. doi: 10.1186/s13104-017-2946-5. PubMed PMID: 29178934; PubMed Central PMCID: PMCPMC5702099.

17.	Alshabanat A, Otterstatter MC, Sin DD, Road J, Rempel C, Burns J, et al. Impact of a COPD comprehensive case management program on hospital length of stay and readmission rates. Int J Chron Obstruct Pulmon Dis. 2017;12:961-71. Epub 2017/03/31. doi: 10.2147/COPD.S124385. PubMed PMID: 28356728; PubMed Central PMCID: PMCPMC5367737.

18.	Moher D, Liberati A, Tetzlaff J, Altman DG, Group P. Preferred reporting items for systematic reviews and meta-analyses: the PRISMA statement. PLoS Med. 2009;6(7):e1000097. Epub 2009/07/22. doi: 10.1371/journal.pmed.1000097. PubMed PMID: 19621072; PubMed Central PMCID: PMCPMC2707599.

19.	Low V, Ben-Shlomo Y, Coward E, Fletcher S, Walker R, Clarke CE. Measuring the burden and mortality of hospitalisation in Parkinson's disease: A cross-sectional analysis of the English Hospital Episodes Statistics database 2009-2013. Parkinsonism Relat Disord. 2015;21(5):449-54. Epub 2015/03/05. doi: 10.1016/j.parkreldis.2015.01.017. PubMed PMID: 25737205.

20.	Tension E, Smink A, Redwood S, Darweesh SKL, Cottle H, van Halteren AD, et al. Proactive and Integrated Management and Empowerment in Parkinson's Disease: designing a new model of care. Parkinson's Disease. 2020 (in press).

21.	Cumpston M, Li TJ, Page MJ, Chandler J, Welch VA, Higgins JPT, et al. Updated guidance for trusted systematic reviews: a new edition of the Cochrane Handbook for Systematic Reviews of Interventions. Cochrane Db Syst Rev. 2019;(10). doi: Artn Ed000142

10.1002/14651858.Ed000142. PubMed PMID: WOS:000493915900002.

22.	Sterne JA, Hernan MA, Reeves BC, Savovic J, Berkman ND, Viswanathan M, et al. ROBINS-I: a tool for assessing risk of bias in non-randomised studies of interventions. BMJ. 2016;355:i4919. Epub 2016/10/14. doi: 10.1136/bmj.i4919. PubMed PMID: 27733354; PubMed Central PMCID: PMCPMC5062054 <http://www.icmje.org/coi_disclosure.pdf> and declare: grants from Cochrane, MRC, and NIHR during the conduct of the study. Dr Carpenter reports personal fees from Pfizer, grants and non-financial support from GSK and grants from Novartis, outside the submitted work. Dr Reeves is a co-convenor of the Cochrane Non-Randomised Studies Methods Group. The authors report no other relationships or activities that could appear to have influenced the submitted work.

23.	Bogner HR, Morales KH, de Vries HF, Cappola AR. Integrated management of type 2 diabetes mellitus and depression treatment to improve medication adherence: a randomized controlled trial. Annals of family medicine. 2012;10(1):15-22. Epub 2012/01/11. doi: 10.1370/afm.1344. PubMed PMID: 22230826; PubMed Central PMCID: PMCPMC3262455.

24.	Egan E, Clavarino A, Burridge L, Teuwen M, White E. A randomized control trial of nursing-based case management for patients with chronic obstructive pulmonary disease. Lippincott's case management : managing the process of patient care. 2002;7(5):170-9. Epub 2002/10/24. PubMed PMID: 12394555.

25.	Kalter-Leibovici O, Freimark D, Freedman LS, Kaufman G, Ziv A, Murad H, et al. Disease management in the treatment of patients with chronic heart failure who have universal access to health care: a randomized controlled trial. BMC medicine. 2017;15(1):90. Epub 2017/05/02. doi: 10.1186/s12916-017-0855-z. PubMed PMID: 28457231; PubMed Central PMCID: PMCPMC5410698.

26.	Lu MC, Guo HR, Livneh H, Lin MC, Lai NS, Tsai TY. The effectiveness of nurse-led case management for patients with rheumatoid arthritis in Taiwan. Int J Clin Pract. 2020;74(2):e13443. Epub 2019/10/23. doi: 10.1111/ijcp.13443. PubMed PMID: 31638719.

27.	Tsuchihashi-Makaya M, Matsuo H, Kakinoki S, Takechi S, Kinugawa S, Tsutsui H. Home-based disease management program to improve psychological status in patients with heart failure in Japan. Circulation journal : official journal of the Japanese Circulation Society. 2013;77(4):926-33. Epub 2013/03/19. PubMed PMID: 23502992.

28.	Ell K, Xie B, Quon B, Quinn DI, Dwight-Johnson M, Lee PJ. Randomized controlled trial of collaborative care management of depression among low-income patients with cancer. J Clin Oncol. 2008;26(27):4488-96. Epub 2008/09/20. doi: 10.1200/JCO.2008.16.6371. PubMed PMID: 18802161; PubMed Central PMCID: PMCPMC2653113.

29.	Steel JL, Geller DA, Kim KH, Butterfield LH, Spring M, Grady J, et al. Web-based collaborative care intervention to manage cancer-related symptoms in the palliative care setting. Cancer. 2016;122(8):1270-82. Epub 2016/03/13. doi: 10.1002/cncr.29906. PubMed PMID: 26970434; PubMed Central PMCID: PMCPMC4828258.

30.	Team RC. A language and environment for statistical computing. R Foundation for Statistical Computing. Vienna, Austria. URL <https://wwwR-projectorg/>. 2017.

31.	Viechtbauer W. Conducting Meta-Analyses in R with the metafor Package. J Stat Softw. 2010;36(3):1-48. PubMed PMID: WOS:000281593200001.

32.	Wickham H. ggplot2: Elegant Graphics for Data Analysis. Use R. 2009:1-212. doi: 10.1007/978-0-387-98141-3. PubMed PMID: WOS:000269437100014.

33.	Katon WJ, Lin EH, Von Korff M, Ciechanowski P, Ludman EJ, Young B, et al. Collaborative care for patients with depression and chronic illnesses. N Engl J Med. 2010;363(27):2611-20. Epub 2010/12/31. doi: 10.1056/NEJMoa1003955. PubMed PMID: 21190455; PubMed Central PMCID: PMCPMC3312811.

34.	Miklavcic JJ, Fraser KD, Ploeg J, Markle-Reid M, Fisher K, Gafni A, et al. Effectiveness of a community program for older adults with type 2 diabetes and multimorbidity: a pragmatic randomized controlled trial. BMC Geriatr. 2020;20(1):174. Epub 2020/05/15. doi: 10.1186/s12877-020-01557-0. PubMed PMID: 32404059; PubMed Central PMCID: PMCPMC7218835.

35.	Callahan CM, Boustani MA, Unverzagt FW, Austrom MG, Damush TM, Perkins AJ, et al. Effectiveness of collaborative care for older adults with Alzheimer disease in primary care: a randomized controlled trial. Jama. 2006;295(18):2148-57. Epub 2006/05/11. doi: 10.1001/jama.295.18.2148. PubMed PMID: 16684985.

36.	Chen Y, Funk M, Wen J, Tang X, He G, Liu H. Effectiveness of a multidisciplinary disease management program on outcomes in patients with heart failure in China: A randomized controlled single center study. Heart and Lung: Journal of Acute and Critical Care. 2018;47(1):24-31.

37.	Connor KI, Cheng EM, Barry F, Siebens HC, Lee ML, Ganz DA, et al. Randomized trial of care management to improve Parkinson disease care quality. Neurology. 2019;92(16):e1831-e42. Epub 2019/03/25. doi: 10.1212/wnl.0000000000007324. PubMed PMID: 30902908; PubMed Central PMCID: PMCPMC6550506.

38.	Gabbay RA, Anel-Tiangco RM, Dellasega C, Mauger DT, Adelman A, Van Horn DH. Diabetes nurse case management and motivational interviewing for change (DYNAMIC): results of a 2-year randomized controlled pragmatic trial. Journal of diabetes. 2013;5(3):349-57. Epub 2013/02/02. doi: 10.1111/1753-0407.12030. PubMed PMID: 23368423; PubMed Central PMCID: PMCPMC3679203.

39.	Gellis ZD, Kenaley B, McGinty J, Bardelli E, Davitt J, Ten Have T. Outcomes of a telehealth intervention for homebound older adults with heart or chronic respiratory failure: a randomized controlled trial. The Gerontologist. 2012;52(4):541-52. PubMed PMID: 365669971.

40.	Hernandez C, Alonso A, Garcia-Aymerich J, Serra I, Marti D, Rodriguez-Roisin R, et al. Effectiveness of community-based integrated care in frail COPD patients: A randomised controlled trial. npj Primary Care Respiratory Medicine. 2015;25 (no pagination)(15022). PubMed PMID: 605195741.

41.	Kroenke K, Theobald D, Wu J, Norton K, Morrison G, Carpenter J, et al. Effect of telecare management on pain and depression in patients with cancer: a randomized trial. Jama. 2010;304(2):163-71. Epub 2010/07/16. doi: 10.1001/jama.2010.944. PubMed PMID: 20628129; PubMed Central PMCID: PMCPMC3010214.

42.	Mertz BG, Dunn-Henriksen AK, Kroman N, Johansen C, Andersen KG, Andersson M, et al. The effects of individually tailored nurse navigation for patients with newly diagnosed breast cancer: a randomized pilot study. Acta oncologica (Stockholm, Sweden). 2017;56(12):1682-9. Epub 2017/08/02. doi: 10.1080/0284186x.2017.1358462. PubMed PMID: 28758822.

43.	Morgan MAJ, Coates MJ, Dunbar JA, Reddy P, Schlicht K, Fuller J. The TrueBlue model of collaborative care using practice nurses as case managers for depression alongside diabetes or heart disease: A randomised trial. BMJ Open. 2013;3 (1) (no pagination)(e002171). PubMed PMID: 368286422.

44.	Riegel B, Carlson B, Glaser D, Romero T. Randomized controlled trial of telephone case management in Hispanics of Mexican origin with heart failure. Journal of cardiac failure. 2006;12(3):211-9. Epub 2006/04/21. doi: 10.1016/j.cardfail.2006.01.005. PubMed PMID: 16624687.

45.	Rose L, Istanboulian L, Carriere L, Price A, Lee L, Rezaie S, et al. Program of integrated care for patients with chronic obstructive pulmonary disease and multiple comorbidities (pic COPD+): A randomized controlled trial. American Journal of Respiratory and Critical Care Medicine Conference: American Thoracic Society International Conference, ATS. 2017;195(no pagination). PubMed PMID: 617710298.

46.	Stoop CH, Nefs G, Pommer AM, Pop VJ, Pouwer F. Effectiveness of a stepped care intervention for anxiety and depression in people with diabetes, asthma or COPD in primary care: A randomized controlled trial. Journal of affective disorders. 2015;184:269-76. Epub 2015/06/30. doi: 10.1016/j.jad.2015.05.063. PubMed PMID: 26118755.

47.	Titova E, Salvesen O, Bentsen SB, Sunde S, Steinshamn S, Henriksen AH. Does an Integrated Care Intervention for COPD Patients Have Long-Term Effects on Quality of Life and Patient Activation? A Prospective, Open, Controlled Single-Center Intervention Study. PloS one. 2017;12(1):e0167887. Epub 2017/01/07. doi: 10.1371/journal.pone.0167887. PubMed PMID: 28060921; PubMed Central PMCID: PMCPMC5218408.

48.	Williams Jr JW, Katon W, Lin EHB, Noel PH, Worchel J, Cornell J, et al. The effectiveness of depression care management on diabetes-related outcomes in older patients. Annals of Internal Medicine. 2004;140(12):1015-24+I-51. PubMed PMID: 38756683.

49.	Wu S, Ell K, Jin H, Vidyanti I, Chou CP, Lee PJ, et al. Comparative Effectiveness of a Technology-Facilitated Depression Care Management Model in Safety-Net Primary Care Patients With Type 2 Diabetes: 6-Month Outcomes of a Large Clinical Trial. Journal of medical Internet research. 2018;20(4):e147. PubMed PMID: 627409483.

50.	Avci IA, Altay B, Cavusoglu F, Cal A, Mumcu N, Eren DC, et al. Evaluation of the Efficacy of the Three-Component Health Care Management Program HEWCOT in Colorectal Cancer Patients Receiving Chemotherapy. Journal of cancer education : the official journal of the American Association for Cancer Education. 2019. Epub 2019/01/04. doi: 10.1007/s13187-018-1461-2. PubMed PMID: 30604384.

51.	Crowley MJ, Edelman D, McAndrew AT, Kistler S, Danus S, Webb JA, et al. Practical Telemedicine for Veterans with Persistently Poor Diabetes Control: A Randomized Pilot Trial. Telemedicine journal and e-health : the official journal of the American Telemedicine Association. 2016;22(5):376-84. Epub 2015/11/06. doi: 10.1089/tmj.2015.0145. PubMed PMID: 26540163.

52.	Johnson JA, Al Sayah F, Wozniak L, Rees S, Soprovich A, Qiu W, et al. Collaborative care versus screening and follow-up for patients with diabetes and depressive symptoms: results of a primary care-based comparative effectiveness trial. Diabetes care. 2014;37(12):3220-6. Epub 2014/10/16. doi: 10.2337/dc14-1308. PubMed PMID: 25315205.

53.	Baker JM, Grant RW, Gopalan A. A systematic review of care management interventions targeting multimorbidity and high care utilization. BMC Health Serv Res. 2018;18(1):65. Epub 2018/02/01. doi: 10.1186/s12913-018-2881-8. PubMed PMID: 29382327; PubMed Central PMCID: PMCPMC5791200.

54.	Vandiver T, Anderson T, Boston B, Bowers C, Hall N. Community-Based Home Health Programs and Chronic Disease: Synthesis of the Literature. Prof Case Manag. 2018;23(1):25-31. Epub 2017/11/28. doi: 10.1097/NCM.0000000000000242. PubMed PMID: 29176341.

55.	Horn S, Hurtig H. The Many Faces of Parkinson's Disease. Cerebrum. 2019;2019. Epub 2020/03/25. PubMed PMID: 32206172; PubMed Central PMCID: PMCPMC7075504.

56.	Lee HM, Koh SB. Many Faces of Parkinson's Disease: Non-Motor Symptoms of Parkinson's Disease. J Mov Disord. 2015;8(2):92-7. Epub 2015/06/20. doi: 10.14802/jmd.15003. PubMed PMID: 26090081; PubMed Central PMCID: PMCPMC4460545.

57.	Timmer MHM, van Beek M, Bloem BR, Esselink RAJ. What a neurologist should know about depression in Parkinson's disease. Pract Neurol. 2017;17(5):359-68. Epub 2017/07/26. doi: 10.1136/practneurol-2017-001650. PubMed PMID: 28739866.

58.	Eggers C, Dano R, Schill J, Fink GR, Hellmich M, Timmermann L, et al. Patient-centered integrated healthcare improves quality of life in Parkinson's disease patients: a randomized controlled trial. J Neurol. 2018;265(4):764-73. doi: 10.1007/s00415-018-8761-7. PubMed PMID: WOS:000428928200004.

**Legend table and figures**

**Table 1:** Description of predefined core elements of case management interventions derived from previous studies [8, 9]

**Table 2:** Main study characteristics

**Table 3:** Specific case management strategies used per study interventions

**Figure 1:** Study flow diagram

**Figure 2:** Forest plot on the effect of case management interventions on feelings of anxiety (random-effects model)

**Figure 3:** Forest plot on the effect of case management interventions on depressive symptoms (random-effects model)

**Figure 4**: Mediators of the effect of case management on reduction of depressive symptoms and feelings of anxiety: hypothetical framework informed by this meta-analysis
