# Supplementary material for: Case management interventions in chronic disease reduce anxiety and depressive symptoms: A systematic review and meta-analysis
Source: PLoS One. 2023 Apr 14;18(4):e0282590. doi: 10.1371/journal.pone.0282590 (PMC10104285; doi:10.1371/journal.pone.0282590)
Supplement: Attachment — Submitted filename: Response to Reviewers.docx [file pone.0282590.s005.docx]

**Response to reviewers**

Thank you for getting back to us for additional information of our manuscript entitled ‘Case management interventions in chronic disease reduce anxiety and depressive symptoms: A systematic review and meta-analysis’. Please find our detailed point-by-point reply below.

**Comment:**

a) Please clarify the sources of funding (financial or material support) for your study. List the grants or organizations that supported your study, including funding received from your institution.

**Response:**

All listed organization provide financial support for doing research, but have not provided financial support specific for the current study.

**Comment:**

b) State what role the funders took in the study. If the funders had no role in your study, please state: “The funders had no role in study design, data collection and analysis, decision to publish, or preparation of the manuscript.”

**Response:**

The funders had no role in study design, data collection and analysis, decision to publish, or preparation of the manuscript.

**Comment:**

c) If any authors received a salary from any of your funders, please state which authors and which funders.

**Response:**

None of the authors has received a salary from the funders.

**Comment:**

d) If you did not receive any funding for this study, please state: “The authors received no specific funding for this work.”

**Response:**

The authors received no specific funding for this work.

**Comment:**

Please include your amended statements within your cover letter; we will change the online submission form on your behalf.

**Response:**

We have included a funding statement in the cover letter.

**Comment:**

In your Data Availability statement, you have not specified where the minimal data set underlying the results described in your manuscript can be found. PLOS defines a study's minimal data set as the underlying data used to reach the conclusions drawn in the manuscript and any additional data required to replicate the reported study findings in their entirety.

**Response:**

The study’s minimal underlying data set has been uploaded as Supporting Information file.

**Comment:**

Please ensure that you refer to Figure 4 in your text as, if accepted, production will need this reference to link the reader to the figure.

**Response:**

Reference to Figure 4 has been added to the Discussion section of the manuscript .

**Comment:**

Please include your tables as part of your main manuscript and remove the individual files. Please note that supplementary tables (should remain/ be uploaded) as separate "Supporting Information" files.

**Response:**

Tables have now been added to the main manuscript file.
